# Metabolic Resistance in Bed Bugs

**DOI:** 10.3390/insects2010036

**Published:** 2011-03-18

**Authors:** Praveen Mamidala, Susan C. Jones, Omprakash Mittapalli

**Affiliations:** 1 Department of Entomology, The Ohio State University, Ohio Agricultural and Research Development Center, Wooster, OH 44691, USA; E-Mail: mamidala.2@osu.edu; 2 Department of Entomology, The Ohio State University, Columbus, OH 43210, USA; E-Mail: jones.1800@osu.edu

**Keywords:** *Cimex lectularius*, insecticides, metabolic resistance, cytochrome P450, glutathione *S*-transferase

## Abstract

Blood-feeding insects have evolved resistance to various insecticides (organochlorines, pyrethroids, carbamates, *etc.*) through gene mutations and increased metabolism. Bed bugs (*Cimex lectularius*) are hematophagous ectoparasites that are poised to become one of the major pests in households throughout the United States. Currently, *C. lectularius* has attained a high global impact status due to its sudden and rampant resurgence. Resistance to pesticides is one factor implicated in this phenomenon. Although much emphasis has been placed on target sensitivity, little to no knowledge is available on the role of key metabolic players (e.g., cytochrome P450s and glutathione *S*-transferases) towards pesticide resistance in *C. lectularius*. In this review, we discuss different modes of resistance (target sensitivity, penetration resistance, behavioral resistance, and metabolic resistance) with more emphasis on metabolic resistance.

## 1. Introduction

Bed bugs (*Cimex lectularius*) are nocturnal hematophagous ectoparasites that have a long-standing association (∼300 BC) with man [1] and during evolution (when human lived in caves *i.e.*, much earlier) these ectoparasites may have switched hosts [2]. Bed bugs preferentially feed on humans, and 70% of individuals have allergic reactions to bed bug bites and experience inflamed welts and severe itching [3]. Scratching then can cause the welts to become infected due to secondary bacterial agents [4,5]. Anxiety, stress, and insomnia are common responses to bed bug infestations [6]. Although there is no definitive evidence that bed bugs transmit blood-borne infectious diseases; these ectoparasites have become an important public health issue that affects all socioeconomic classes [7].

Bed bugs were extremely common pests in the United States prior to World War II. However, the repetitive use of insecticides with long-lasting residual, such as **d**ichloro**d**iphenyl**t**richloroethane (DDT) (an organochlorine), organophosphates, and carbamates, drastically reduced the bed bug population [8]. Since the late 1990s, there has been a worldwide resurgence of bed bugs, particularly in developed countries including Eastern Asia, Europe, Australia, and North America [9–11]. Some of the important factors attributed to the sudden resurgence of bed bugs include increased domestic and international travel (especially from areas where bed bugs remained common); increased commerce involving used furniture and other household items; the shift from broad-spectrum insecticides to narrow control tactics; and the development of insecticide resistance in bed bugs [6,7,9,10,12-17]. Insecticide resistance (DDT and permethrin) has been largely attributed to changes in the bed bugs' gene structure, *i.e.*, point mutations [18] and upregulation of metabolic genes [19].

## 2. Resistance

According to the World Health Organization [20] *resistance* is defined as “the inherited ability of a strain of some organism to survive doses of a toxicant that would kill the majority of individuals in a normal population of the same species.” During the course of evolution, an insect species attains resistance to overcome the insecticidal effects of synthetic chemicals or natural toxins and hence allow for its survival.

To date, various groups of insects have been shown to develop resistance towards insecticides; these include 56% crop pests; 37% medical and veterinary pests (mosquitoes, ticks, *etc.*) and 5% beneficial species [21]. Insects develop resistance to various chemicals through four different modes: target site resistance, behavioral resistance, penetration resistance, and metabolic resistance. Target site resistance and behavioral resistance have been well studied for many insects, whereas metabolic resistance and penetration resistance often have been neglected or overlooked. In this review of all four modes of resistance, we focus on the *modus operandi* of metabolic resistance in bed bugs. We also highlight potential control mechanisms through silencing genes involved in detoxification.

## 3. Target Site Resistance

In general, insecticides react with certain target sites (acetylcholinesterases, gamma aminobutyric acid-gated chloride channels, *etc.*) in the organism thereby inhibiting their enzymatic activity. However, some blood-feeding insects are thought to have evolved resistance (insensitivity) to organophosphate and carbamate insecticides via a mechanism known as target site insensitivity [22]. For example, studies on the mosquitoes, *Anopheles albimanus, Culex pipiens pipiens,* and *Culex quinquefasciatus,* revealed alterations in the acetylcholinesterase genes, which in turn reduced the binding efficiency with insecticides thereby overcoming insecticidal effects [23].

Voltage-gated sodium channels play an important role in the rising phase of action potentials in neurons [24]. Voltage-gated sodium channels were first cloned from *Drosophila melanogaster* and since then several studies have used this information to compare similar sequences among susceptible and resistant insect populations in order to determine possible resistance due to mutations [25–29]. Pyrethroids are well known to exert insecticidal effects by altering the function of voltage-sensitive sodium channels in nerve membranes of many insect species. It has been documented that pyrethroid resistance stems from point mutations in the voltage-gated sodium channels [24]. As a result, this feature has been linked to knockdown resistance, commonly termed *kdr*. The *Kdr* phenotype results from point mutations in coding sequences of voltage gated sodium channels (insect nervous system) thereby resulting in resistance to DDT and pyrethroids [30]. *Kdr* mutations are well documented in various blood-feeding insects including *Anopheles gambiae -* L1014F; *Cu. pipiens -* L1014F and L1014S; *Pediculus capitis -* T929I and L932F; and *C. lectularius -* V419L and L925I [31–34].

Resistance to DDT and pyrethroids by bed bugs is suspected to result from point mutations in voltage-gated sensitive channels [34]. In a recent study, Zhu *et al.* [15] further suggested that the widespread distribution of bed bugs across North America can be attributed to target-site sensitivity of the voltage-gated sodium channel α-subunit gene among the populations scanned. However, it will be interesting to decipher whether pyrethroid resistance in bed bugs is attributable solely to mutations in voltage-gated channels, increased detoxification strategies, or a combination of the aforementioned factors.

## 4. Behavioral Resistance

Behavioral resistance, the ability to survive insecticide applications through behavioral adaptation, is much less common than physiological resistance. Behavioral responses of arthropod crop pests as well as medical, veterinary, and urban pests have been reviewed by numerous authors [35–41]. These reports cover a wide range of taxonomic groups, particularly Acari, Coleoptera, Lepidoptera, Orthoptera, Hemiptera, and Diptera.

Behavioral resistance is well documented in some blood-feeding insects [42]. However, research is scant on behavioral resistance in bed bugs. In a recent study, it was shown that bed bugs avoided resting on deltamethrin-treated filter paper, a kind of behavioral resistance wherein the insect keeps away from the insecticide [43].

Despite the many studies documenting behavioral responses to insecticides, little is known at the functional level about the genes involved in behavioral traits associated with toxins compared to the genes that metabolize them. However, recent high throughput technologies have allowed the identification of putative candidate genes influencing insect behavior and have simultaneously furthered the understanding of the physiology-driven molecular mechanisms influencing insecticide/allelochemicals resistance [44]. Elucidating the genetic basis of behavioral resistance is crucial to an understanding of insect adaptation to toxins.

## 5. Penetration Resistance

Many insects overcome the effects of insecticides through decreased cuticular penetration, which is a well-documented phenomenon [45–47]. In simple terms, the thicker the cuticle (with higher protein and lipid content), the less able it is to absorb a toxin. This mode of resistance could include the development of barriers in resistant insects. Penetration resistance is usually associated with other forms of resistance and thus this trait could intensify the effect of the resistance mechanisms. Interestingly, a high number of cuticular domains were identified in our recent 454 pyrosequencing of bed bugs [19]. Morphological (scanning electron microscopy studies) and biochemical (characterization of proteins involved in detoxification) comparisons of cuticular proteins among resistant and susceptible bed bugs may provide more clues to bed bug resistance to insecticides.

## 6. Metabolic Resistance

Metabolic resistance is basically the biochemical transformation of a toxin, wherein the toxic compound is transformed into a less toxic form [48]. Biotransformation reactions are classified as Phase I or Phase II based on their mode of action on toxic compounds. The Phase I reactions include oxidation, reduction, and hydrolysis; they are involved in detoxification of xenobiotics. The Phase II reaction is a process of biosynthetic conjugation wherein the conjugative enzymes bind with toxins or primary products of Phase I and convert them into more water soluble derivatives, which are excreted from the body. Cytochrome P450 monoxygenases (P450s) are Phase I metabolic enzymes capable of oxidizing endogenous and exogenous compounds by oxidation or other related reactions [48]. Glutathione *S*-transferase (GST) is a Phase II metabolizing enzyme that catalyzes the reaction of reduced glutathione to compounds bearing electrophilic sites [49].

The detoxification of synthetic chemicals by insects is mainly associated with cytochrome P450s and with large multigene families such as esterases, oxidases, and transferases [50–55]. Among these detoxifying enzyme systems, cytochrome P450s are involved in the metabolism of pyrethroids; GSTs detoxify organochlorine compounds; and esterases and oxidases act primarily on carbamates [56-58]. The putative insect genes involved in metabolic resistance to various insecticides are summarized in Table 1.

### 6.1. Cytochrome P450s

Cytochrome P450 (P450s/CYPs; **P**igment with **450** nm absorption) enzymes are the largest superfamily of proteins found in all living organisms. Thus far about 59 CYP families (338 subfamilies) have been identified among 89 insect species [59]. These CYPs are distributed among four CYP clades (CYP2, CYP3, CYP4 and mitochondrial CYP) [60]. The unique features of P450 proteins (genetic diversity, broad substrate specificity, catalytic versatility) have made them capable of dealing with almost all types of insecticides [48]. Though CYPs exhibit high diversity among their amino acid sequences, it is reported that these proteins share certain structural sequence conservation in the heme-binding loop (Phe-X-X-Gly-X-Arg-X-Cys-X-Gly), K-helix (Glu-X-X-Arg), and central part of the I-helix (Ala/Gly-Gly-X-Asp/Glu-Thr-Thr/Ser) [48].

The P450s, in particular CYP4, CYP6 and CYP9, are well studied and have been characterized in many insects wherein they play a vital role in xenobiotic metabolism [56,61–65]. In general, P450 enzymes bind molecular oxygen [S+(NADPH+H^+^)+O_2_ − S(O)+ NADP^+^+ H_2_O +; wherein S is the substrate] and receive electrons from NADPH leading to formation of water [66]. Recent genome sequences of several blood-feeding insects revealed the occurrence of ∼37–102 P450 genes probably each encoding for different P450 enzymes [67–70]. The human body louse, *Pediculus humanus*, had the smallest genome with a decreased number of CYPs (37) (Table 1), which might be due to this species' to show possession low exposure to various insecticides [67,71].

The CYP6 family has been well documented as being involved in insecticide resistance in blood-feeding insects [65]. The first insect P450 reported to be involved in detoxification was CYP6A1 of the house fly, *Musca domestica* [61]. Since then several CYP6 members have been identified and well characterized as to their involvement in xenobiotic metabolism [72]. In a recent study, overexpression of a brain-specific CYP6BQ9 of *Tribolium castaneum* followed by knock-out experiments revealed its major role in deltamethrin resistance [73]. In another study, CYP9M10 of *Cu. quinquefasciatus* was shown to be involved in pyrethroid detoxification [74].

After the successful completion of the genome sequence of *A. gambiae*, much research was focused on deciphering the involvement of detoxifying enzymes in insecticide resistance; initial findings revealed that resistant strains had higher expression of CYP4C27, CYP4H15, CYP6Z1, CYP6Z2, and CYP12F1 [75]. Further, to confirm candidate CYPs (CYP6Z1, CYP6Z2) in DDT metabolism, computational molecular models of these two proteins with and without DDT docking revealed CYP6Z1 as a likely enzyme involved in insecticidal resistance [76]. Similar molecular modeling experiments in bed bugs may shed light on designing novel inhibitors for effective control.

It is interesting to note that expression patterns of P450s in insects vary with tissue, development, and sex [77]. In our recent study, we reported high transcript levels for CYP9 in early instars of the pesticide-exposed population of bed bugs [16]. Further, on-going studies of the bed bug revealed a higher expression (mRNA levels) for one of the cytochrome P450s in the cuticle of pesticide-exposed populations compared to pesticide-susceptible populations. These preliminary results perhaps indicate a role of CYPs in the cuticle of the bed bug (Mittapalli *et al.*, unpublished data). However, the expression of P450s among male and female bed bugs has yet to be determined. In *Ips paraconfusus*, few P450s (CYP9T1, CYP4AY1, CYP4BG1) were involved in synthesis of the male-specific aggregation pheromone [78].

### 6.2. Glutathione S-Transferases

Glutathione *S*-transferases (GSTs) are one the major components of detoxification pathways in living organisms. GSTs play an important role in xenobiotic metabolism through catalysis of redox and conjugation reactions, which in turn facilitates the solubility of compounds, furthering the excretion of these toxic compounds from the system. Several GSTs identified in blood-feeding insects have shown higher expression when the insects were exposed to various insecticides [79–81]. While feeding, insects usually face a plethora of reactive oxygen species (ROS), during which GSTs are thought to play an important role in protecting the cells against oxidative stress [82].

GSTs are classified into three groups based on their cellular localization: cytosolic, microsomal, and mitochondrial. Blood-feeding insects are thought to possess both cytosolic and microsomal GSTs [83]. The GST family in insects encodes for a diverse set of proteins including Delta, Epsilon, Sigma, Theta, Omega, and Zeta. Among these classes, Delta and Epsilon have been reported to be highly expressed in insects that encounter insecticides.

In our recent study, we found a high occurrence (14) of GSTs in the transcriptomic data of bed bugs [19]. qPCR analysis of a candidate GST (Delta/Epsilon) revealed higher expression in the early and late instar stages of the pesticide-exposed populations as compared to pesticide- susceptible populations. Intriguingly, no significant difference in expression of GST transcript levels was observed among the adult bed bug populations [19].

### 6.3. Carboxylesterases

Carboxylesterases are multifunctional proteins primarily involved in detoxification of insecticides and pheromone degradation as reported in many insect pests [84,85]. In particular, these enzymes are involved in degradation of organophosphates, carbamates, and pyrethroids. Internal genomic alterations (increased copy number/mutations in coding sequences) among carboxylesterases are one of the main factors underlying the development of insecticide resistance [48].

#### 6.3.1. Endosymbionts

Microorganisms are ubiquitous in nature and play an important role in the successful adaptation of insects. Insects' microbiota are involved in the synthesis of essential amino acids and vitamins, digestive processes, reproduction, and detoxification of harmful compounds to safer products within the insect host [86–93]. Recent studies on bacteria associated with nutrition in bed bugs revealed the role of *Wolbachia* in synthesis of B vitamins. Intriguingly, elimination of these microbes resulted in retarded growth and sterility of bed bugs [93]. Much research now has focused on the influence of microbial endosymbionts in the host's nutritional homeostasis; however, whether these endosymbionts are involved in detoxification of insecticides remains elusive.

#### 6.3.2. Heme Detoxification

Heme is involved in biological reactions such as oxygen transport and respiration. Bed bugs require blood meal during all developmental stages. Blood heme (bound to hemoglobin) is a toxic molecule due to its ability of generating (ROS), which damages and disrupts the phospholipid bilayer of cell membranes [94]. The enzymatic systems that could potentially deal with the ROS produced include superoxide dismutase (SOD), catalase (CAT), glutathione peroxidase (GPX), glutathione reductase (GR), *etc*. [95]. SOD dismutates the superoxide radical to stable hydrogen peroxide and oxygen, whereas CAT transforms hydrogen peroxide into water and oxygen, and GPX targets hydroperoxides. Blood-feeding insects seem to have adapted against heme toxicity during the course of evolution [96], but scarce knowledge exists in bed bugs. Pursuing the latter studies would allow the identification of potential metabolic targets that may influence growth and development in the bed bug.

#### 6.3.3. RNAi

RNA interference (RNAi) has revolutionized the fields of medicine and agriculture in the recent past. Besides its use in functional studies, RNAi can be used as a potential tool for developing novel control measures for insect pests. Such utility of RNAi through feeding and transformation technology has initiated more studies for pest control [97,98]. Since then RNAi studies have been performed in several insects of agricultural importance and in a few blood-feeding insects [99]. Silencing or knocking off candidate genes involved in insecticide resistance of bed bugs could also provide potential avenues for the development of newer/more efficient control strategies.

## 7. Conclusions

Lessons learned to date clearly indicate the complex nature of bed bugs' resistance to pesticides. As of now, resistance in bed bugs appears to be attributed toward: (i) point mutations in the nervous system genes (e.g., voltage-gated sensitive channels); (ii) differential expression of detoxification genes (e.g., cytochrome P450s and GSTs) and (iii) morphological traits such as thickened cuticle in resistant populations compared to susceptible ones. Future studies aimed at deciphering genome-wide (global) expression patterns among susceptible and resistant bed bug strains and gene knock out experiments may provide insights into the genetic basis for insecticide resistance in bed bugs. Further, comparative metagenomics of bed bugs' microbiota may also unravel the role of these microorganisms in host resistance towards synthetic chemicals.

## Figures and Tables

**Table 1:** Mode of resistance and number of cytochrome P450s, carboxylesterases, esterases, and transferases among the genomes of blood-feeding insects^19,66-69^.

**Mode of Resistance**	***Pediculus humanus***	***Anopheles gambiae***	***Culex quinquefasciatus***	***Aedes aegypti***	Cimex lectularius*
	**TSR**	**MR & TSR**	**MR & TSR**	**MR & TSR**	**MR & TSR**
P450	37	106	172	158	73#
CES	not reported	25	47	30	-
EST	17	15	17	19	-
TRA	13	31	37	32	14#

MR = metabolic resistance; TSR=target sensitivity resistance; P450 = cytochrome P450s; CES = carboxylesterases; EST = esterases; TRA = transferases; # = occurrences;

* as per 454 pyrosequencing data, not complete genome of the bed bug [19].
